# MoS_2_/HfO_2_ Heterostructure Interface Engineering for Stable and Reproducible Resistive Switching in Ag/HfO_2_/TiN Memristors for Intelligent Sensor Nodes

**DOI:** 10.3390/s26217035

**Published:** 2026-11-06

**Authors:** Ya-Zhou Zhou, Le-Tian Fang, Zhen-Nan Zhou, Nan Zhou, Yun Wang, Fei Shan

**Affiliations:** 1 School of Mechanical Engineering, Jiangsu University, Zhenjiang 212013, China; 2 College of Ocean, Jiangsu University of Science and Technology, Zhenjiang 212000, China; 3 Suzhou Yuanzi Intelligent Technology Co., Ltd., Suzhou 215000, China

**Keywords:** memristor, MoS_2_, HfO_2_, intelligent sensing, robotic perception, near sensor memory, interface engineering, oxygen vacancy

## Abstract

Resistive switching devices are attractive candidates for nonvolatile memory and sensory information storage in intelligent sensor nodes, but their use near sensors remains limited by switching variability and resistance state instability. In this work, a two-dimensional MoS_2_ interlayer is introduced into an Ag/HfO_2_/TiN memristor stack to form a MoS_2_/HfO_2_ heterostructure device, and its electrical characteristics are compared with an Ag/HfO_2_/TiN control device fabricated under the same oxide deposition and annealing conditions. The heterostructure device exhibits well-defined and reproducible bipolar resistive switching with an on/off ratio of up to approximately 10^3^, together with markedly improved cycle-to-cycle overlap of the current-voltage characteristics, whereas the switching window of the control device narrows and eventually closes after repeated sweeps. X-ray photoelectron spectroscopy of the O 1s core level shows that the oxygen-vacancy-related component decreases from 18.31% to 5.12% of the fitted spectral envelope after introduction of the MoS_2_ interlayer, while the lattice-oxygen component remains essentially unchanged. This selective reduction is attributed to interfacial passivation of electrically active vacancies rather than to bulk oxygen extraction, and it cannot be explained by signal attenuation of the buried oxide under the MoS_2_ overlayer. Retention measurements show that the heterostructure device maintains stable high- and low-resistance states over the measured 10^3^ s window, while the high-resistance state of the control device decays by orders of magnitude within the same period. The switching polarity, the correlation between vacancy chemistry and switching uniformity, and the stability of the ruptured-filament state are consistent with an interface-guided valence-change mechanism, although a minor contribution of Ag-ion migration cannot be excluded. These results indicate that MoS_2_/HfO_2_ heterojunction engineering is an effective route toward mitigating variability and retention degradation in oxide-based memristors for intelligent sensing hardware. The measured resistance window, repeatable switching, and state retention are relevant to local storage of sensor features, calibration coefficients, and adaptive thresholds in robotic perception. The present work establishes the memory component of such sensor nodes and does not claim direct transduction or a complete robotic sensing system.

## 1. Introduction

Memristive devices, also referred to as resistive switching devices, are two-terminal circuit elements whose resistance depends on the history of the applied voltage or current. Their ability to emulate synaptic plasticity in hardware makes them promising candidates for overcoming the von Neumann bottleneck in data-intensive computing [1,2,3]. With the rapid development of artificial intelligence and sensing technologies, application scenarios such as intelligent robots, unmanned aerial vehicles, and automated guided vehicles impose increasingly stringent requirements on smart hardware in terms of power consumption, integration density, and real-time processing capability [4,5,6]. In neuromorphic architectures, memristors can implement long-term potentiation and depression and support online learning in spiking neural networks [7,8,9]. For intelligent machines that rely on multimodal sensing, memristive devices can in principle enable real-time path planning, sensor-data processing, and adaptive navigation under tight energy and area budgets [10,11,12,13,14]. Realizing these prospects requires memory elements that switch reproducibly and retain their programmed states reliably, and these two requirements remain difficult to satisfy simultaneously.

An intelligent sensing system contains both transducers and circuits that store and process the resulting information. A pressure, strain, or optical sensor converts a physical stimulus into an electrical signal, whereas an adjacent nonvolatile memory element can retain a calibration coefficient, an adaptive threshold, or a locally extracted feature after the stimulus has ended. Near-sensor and in-sensor computing architectures use this proximity to reduce data transfer, latency, and energy consumption [15]. This distinction is important for the present work because the memristor is not assigned the function of direct transduction. Its proposed role is to provide local state storage within a broader sensing chain.

Robotic tactile perception provides a concrete application context. Electronic skins and tactile arrays produce distributed measurements that support contact detection, object manipulation, and feedback control in intelligent robots [16]. Artificial afferent nerves and neuromorphic sensorimotor loops demonstrate the value of combining sensory encoding with local processing and actuation [17,18]. Such architectures require reliable memory near the sensing location, particularly when thresholds or features must remain available between sensing events. Reproducible switching, a distinguishable resistance window, and stable retention are therefore relevant device-level requirements for the memory component of robotic sensing nodes.

A typical resistive switching device stores information as a reversible transition between a high-resistance state (HRS) and a low-resistance state (LRS), driven by physical changes in the active layer such as the formation and rupture of conductive filaments composed of mobile ions or vacancies [19,20,21,22,23,24]. Resistive switching has by now been demonstrated in a wide range of transition-metal oxides. Representative examples include NiO, for which stable switching junctions have been realized on single nanowires [25], and Fe_3_O_4_, for which oxide hetero-nanocrystals with well-controlled interfaces exhibit uniform switching with a switching probability of approximately 90% [26], in addition to the large family of binary and complex oxides surveyed in recent reviews [27]. These material systems share the filamentary switching phenomenology while differing widely in the achievable device metrics. In filamentary oxide devices, the stochasticity of filament nucleation and growth is the primary origin of cycle-to-cycle and device-to-device variability, while interfacial degradation and uncontrolled oxygen vacancy redistribution limit the stability of the programmed states [28,29]. Mitigating this variability and improving resistance-state retention are therefore essential steps toward reliable device operation.

Two-dimensional materials, including transition metal dichalcogenides such as WSe_2_ and MoS_2_, van der Waals insulators such as h-BN, and related layered compounds, offer atomically flat and dangling-bond-free interfaces together with a rich defect chemistry, and thereby provide new opportunities for controlling filament formation in memristors [30,31,32,33]. In MoS_2_-based devices, conductive filaments have been shown to nucleate preferentially along sulfur-vacancy-rich paths, layer edges, and van der Waals gaps, as revealed by in situ transmission electron microscopy [33,34] and by density-functional and ab initio modeling [35,36]. The MoS_2_/HfO_2_ materials pair is of particular interest. The HfO_2_/MoS_2_ interface has been characterized in detail in the context of high-k gate-dielectric integration, including its band alignment [37], and MoS_2_/HfO_2_ heterostructures have been exploited in optoelectronic switching devices [38]. In the memory context, inserting a MoS_2_ layer into a conducting-bridge device has been shown to regulate metal-ion filaments and improve switching uniformity [39].

Hafnium oxide is one of the most mature resistive switching materials, owing to its high dielectric constant and its well-understood vacancy-mediated switching behavior [40,41,42]. Well-performing Ag/HfO_2_/TiN-type devices reported in the literature generally rely on optimized current-compliance and electroforming conditions, under which both oxygen-vacancy and Ag-related filaments can contribute to the switching [43,44]. Interface and multilayer engineering of HfO_2_, for example, stacking an oxygen-vacancy-rich Al-ZnO interlayer between HfO_2_ layers, has proven effective in enlarging the switching window and improving retention [45], and work on HfO_2_-based ferroelectric films likewise highlights the decisive role of oxygen-vacancy migration and heterointerfaces in governing the switching characteristics [46]. What remains less explored is how a two-dimensional interlayer reshapes the vacancy landscape of the oxide switching layer itself, that is, interface-induced defect modulation rather than simple filament confinement, and how this modulation correlates with switching uniformity and resistance-state retention. Addressing this question requires a direct comparison between heterostructure and oxide-only devices fabricated under identical conditions, combined with chemical-state analysis of the switching layer.

In the present work, the influence of a MoS_2_/HfO_2_ heterostructure on the electrical behavior of Ag/MoS_2_/HfO_2_/TiN/SiO_2_/Si memristors is investigated systematically, using an identically processed Ag/HfO_2_/TiN/SiO_2_/Si device as the control. The MoS_2_ interlayer provides an atomically smooth van der Waals interface with HfO_2_ that reduces the interfacial defect density and regulates vacancy migration, while HfO_2_ remains the switching layer hosting the resistive transition. By correlating current-voltage characteristics, retention behavior, and X-ray photoelectron spectroscopy (XPS) of the O 1s core level, it is shown that the heterostructure suppresses the population of electrically active oxygen vacancies and improves switching reproducibility and state stability. The electrical behavior is characterized by quasi-static current-voltage sweeps and retention measurements. In contrast to earlier MoS_2_-inserted conducting-bridge devices, in which the two-dimensional layer modulates metal filaments [39], the polarity and chemical evidence presented here are consistent with an interface-guided valence-change mechanism, without excluding a secondary Ag-ion contribution, which distinguishes the present device concept from both MoS_2_-only memristors and conventional Ag/HfO_2_/TiN devices.

## 2. Experiment

**Figure 1:**
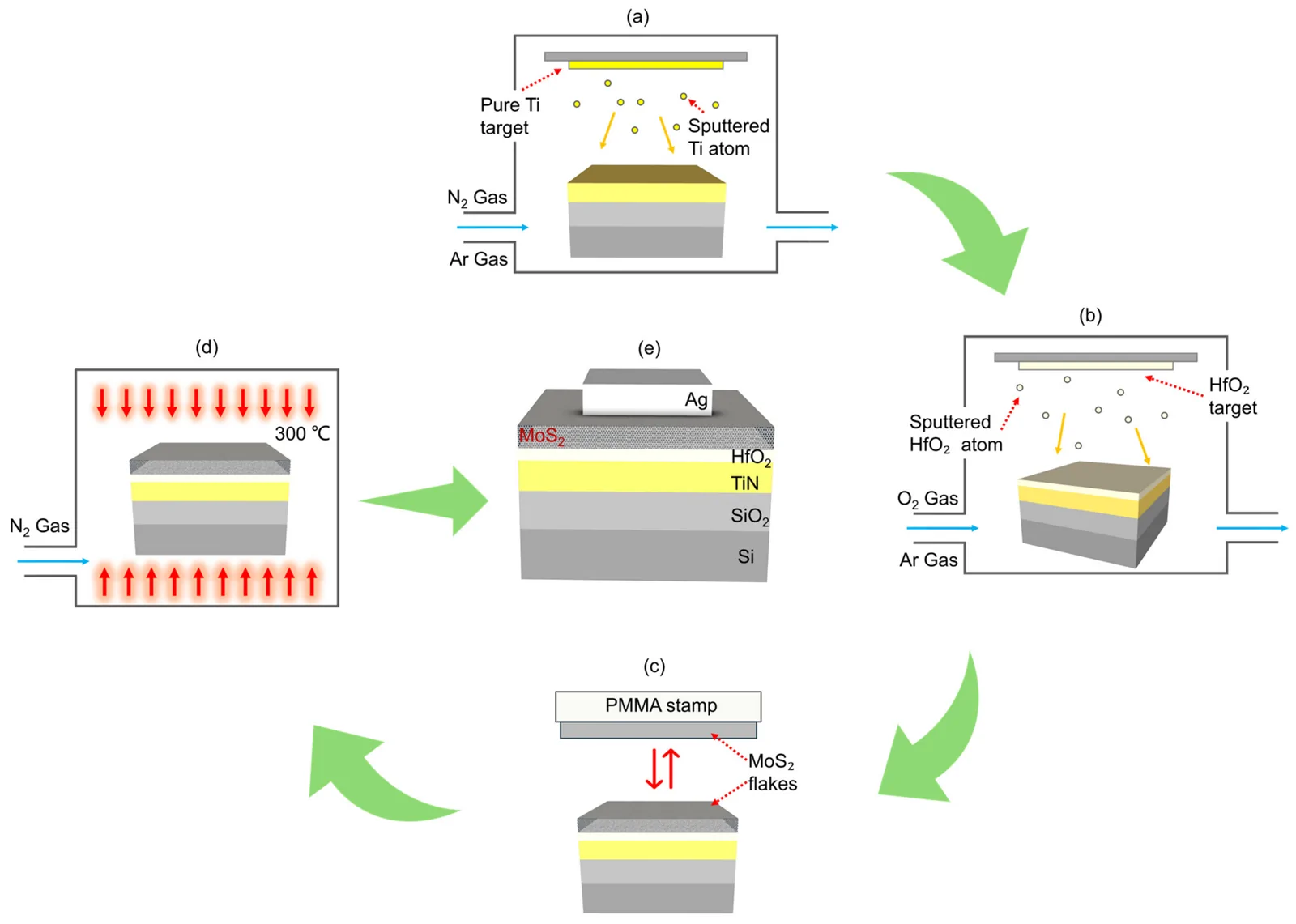
Schematically illustrates the fabrication process flow of the memristor device employing a MHH stacked structure. (**a**) A TiN bottom electrode is deposited by radio-frequency magnetron sputtering. (**b**) A HfO_2_ resistive switching thin film is sputter-deposited using a high-purity HfO_2_ target, followed by annealing at 300 °C for 5 min. (**c**) The MHH structure is formed via mechanical exfoliation and patterned transfer of MoS_2_. (**d**) Annealing is performed under an N_2_ atmosphere at 300 °C for 30 min. (**e**) The final memristor device with the stacked MHH structure is obtained.

Figure 1 presents the fabrication methodology and structural architecture of the memristor, which employs the layer stack Ag/MoS_2_/HfO_2_/TiN/SiO_2_/Si (denoted MHHM). First, a SiO_2_/Si substrate was cleaned and dried, after which a 50 nm-thick TiN bottom electrode was deposited by radio-frequency (RF) magnetron sputtering (The1-MSS2) at a working power of 350 W and a chamber pressure of 6 mTorr (Figure 1a). Subsequently, a 7–8 nm HfO_2_ resistive switching film was sputtered at room temperature from a high-purity HfO_2_ target at 50 W and 6 mTorr, with Ar and O_2_ flow rates of 10 and 0.2 sccm, respectively (Figure 1b), followed by annealing in a vacuum oven at 300 °C for 5 min. HfO_2_ films sputtered at room temperature and annealed at such moderate temperatures are generally expected to remain predominantly amorphous, because crystallization of HfO_2_ typically requires a substantially higher thermal budget [40,41,42]. MoS_2_ flakes were then obtained by mechanical exfoliation from bulk crystals, picked up with a poly(methyl methacrylate) (PMMA) stamp, and aligned and transferred onto the HfO_2_/TiN/SiO_2_/Si substrate to form the heterojunction (Figure 1c). This exfoliation/transfer step is necessarily ex situ, so the process flow contains a vacuum break with ambient exposure between the oxide deposition and the MoS_2_ transfer. The transferred flakes are few-layer MoS_2_, as identified from their optical contrast during the transfer step, and only flakes with lateral dimensions larger than the 50 μm electrode diameter were selected under an optical microscope, so that the MoS_2_ flake fully covers the top-electrode area of each device. An additional annealing step was performed in a furnace under N_2_ atmosphere at 300 °C for 30 min to improve the MoS_2_/HfO_2_ interface quality and mitigate transfer-induced residues (Figure 1d). Finally, a ~50 nm-thick Ag top electrode was deposited to complete the device (Figure 1e), defining a circular contact with a diameter of 50 μm (contact area as 2.0 × 10^−3^ mm^2^). It should be noted that this additional N_2_ annealing step introduces a difference in thermal budget between the MHHM and the control devices described below, and its possible contribution to the observed electrical and chemical differences is therefore considered explicitly in the discussion.

To isolate the influence of the MHH structure, a control device with the stack Ag/HfO_2_/TiN/SiO_2_/Si (denoted HM) was fabricated under the same TiN and HfO_2_ deposition conditions and the same post-deposition annealing of the HfO_2_ film, differing only in the absence of the MoS_2_ transfer and its associated N_2_ annealing step. Current–voltage (I–V) characteristics were measured with a semiconductor parameter analyzer (4200-SCS, Keithley, Solon, OH, USA) by sweeping the voltage applied to the Ag top electrode between −1 V and +1 V, with the TiN bottom electrode grounded. Logarithmic I–V analysis and XPS of the O 1s core level (Thermo Scientific Nexsa, Hillsboro, OR, USA, monochromatic Al Kα source with hν = 1486.6 eV) were used to examine the transport behavior and the oxygen chemical states, respectively. The XPS spectra were charge-referenced to the C 1s line of adventitious carbon at 284.8 eV and fitted with Gaussian–Lorentzian line shapes after Shirley background subtraction. The electrical characterization is limited to quasi-static I–V sweeps and retention tests, and accordingly the analysis centers on switching uniformity, resistance-state stability, and defect modulation.

## 3. Results and Discussion

**Figure 2:**
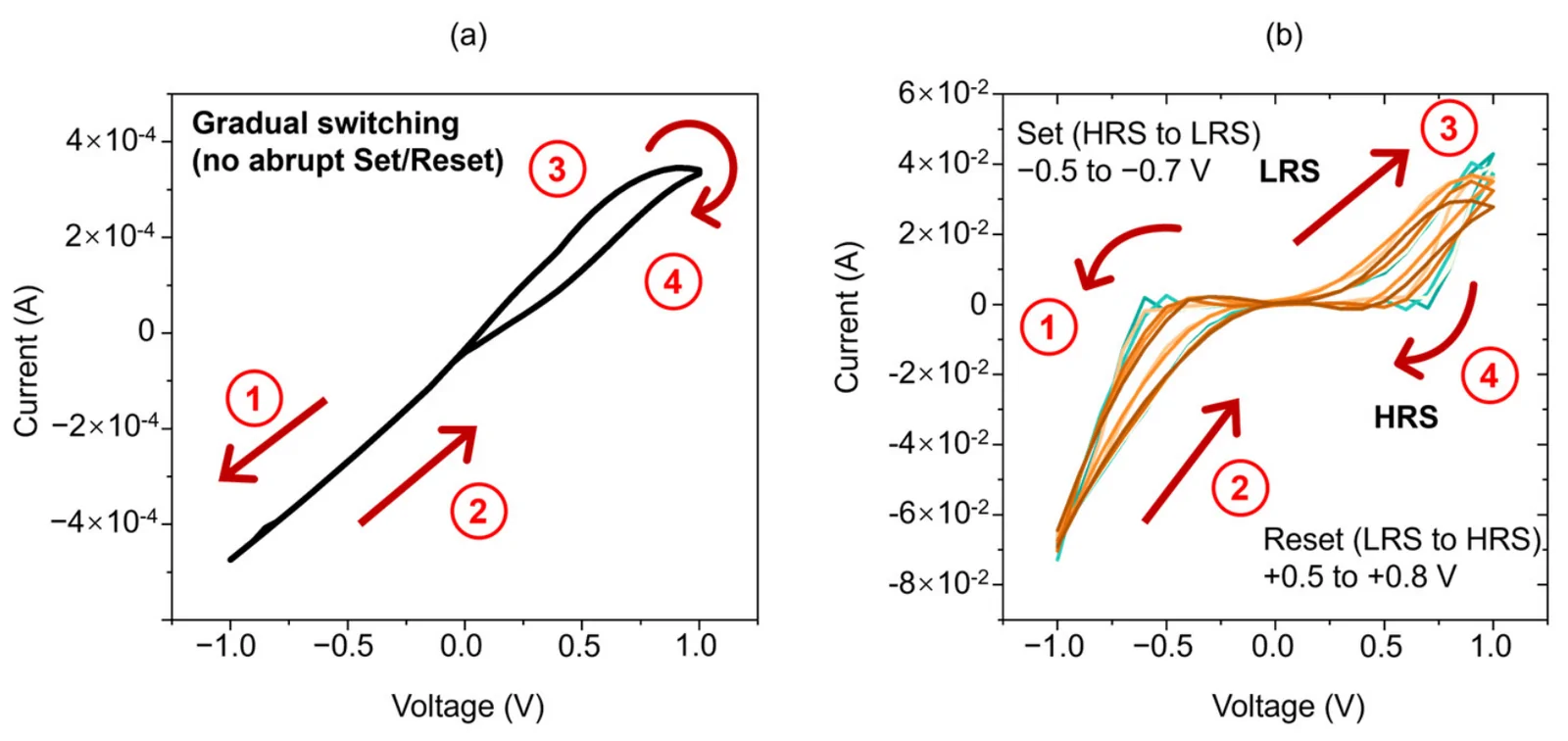
Current response of (**a**) HM and (**b**) MHHM devices under single and multiple voltage sweep cycles ranging from +1 V to −1 V. Arrows indicate the voltage-sweep direction, and the high-resistance-state (HRS) and low-resistance-state (LRS) branches and the Set (HRS → LRS, under negative bias) and Reset (LRS → HRS, under positive bias) transitions are explicitly marked.

Figure 2 compares the current-voltage characteristics of the HM and MHHM devices measured under cyclic voltage sweeps between −1 V and +1 V applied to the top electrode. The polarity convention is defined at the outset. In the present configuration, the Set transition from the HRS to the LRS occurs during the negative voltage ramp, and the Reset transition from the LRS to the HRS occurs during the positive voltage ramp. The MHHM device, shown in Figure 2b, exhibits a broad and reproducible hysteresis window. Upon sweeping from 0 V to −1 V, the current rises sharply near approximately −0.5 V to −0.7 V, and during the subsequent positive ramp the device remains in the LRS until the current drops abruptly near approximately +0.5 V to +0.8 V. The cycle-to-cycle distributions extracted from the repeated sweeps of Figure 2b give Set voltages between −0.7 V and −0.5 V, Reset voltages between +0.5 V and +0.8 V, and peak LRS currents between 2 × 10^−2^ A and 4 × 10^−2^ A. The HM control device, shown in Figure 2a, also displays hysteresis during the first positive sweeps, but its window is narrower and less well defined, with a gradual rather than abrupt current transition. After repeated sweeps, the hysteresis of the HM device closes, indicating that stable bipolar switching is not sustained under the present measurement conditions.

For the MHHM device, the Set threshold fluctuates between approximately −0.7 V and −0.5 V across cycles, the Reset threshold varies between approximately +0.5 V and +0.8 V, and the peak LRS current fluctuates within 2 × 10^−2^ A to 4 × 10^−2^ A. This residual spread is attributed to the nanoscale inhomogeneity of the initial vacancy distribution and of the local electric field in the HfO_2_ layer. Filaments nucleated in vacancy-rich regions require a smaller voltage magnitude, whereas vacancy-sparse regions demand a larger one. Concerning the switching direction, oxygen vacancies carry an effective positive charge. Under a negative Set bias applied to the Ag top electrode, the vacancies therefore drift toward the negatively biased top electrode, that is, toward the MoS_2_/HfO_2_ interface, where they accumulate and nucleate a filament that propagates toward the TiN bottom electrode. Under the positive Reset bias, the vacancies are driven back toward the bottom electrode, and the filament ruptures near its weakest point, typically close to the interface region where it is thinnest. This polarity assignment is fully consistent with the observed Set under negative bias and Reset under positive bias in Figure 2b. The HM device, by contrast, shows no distinct switching event under negative bias. This behavior is interpreted as follows. In the absence of the MoS_2_ interlayer, the filaments formed during the positive sweep rupture incompletely and leave residual conductive paths. The subsequent negative sweep therefore probes a partially connected filament network rather than a well-defined HRS, which produces the overlapping and hysteresis-free curves visible in Figure 2a. With repeated cycling, random vacancy redistribution and interfacial trapping further erode the window, consistent with the eventual closure of the hysteresis loop.

**Figure 3:**
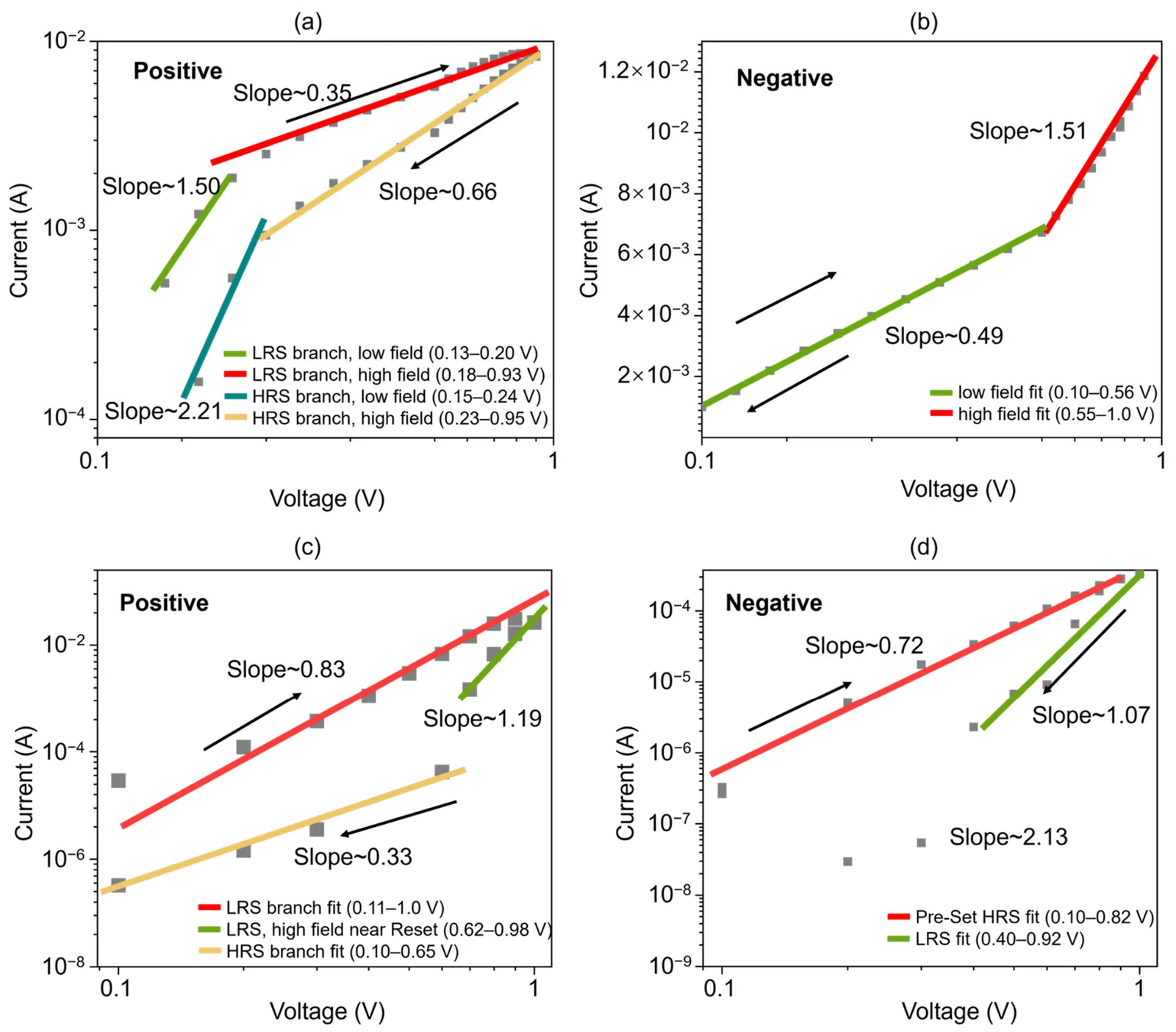
Current response of (**a**,**b**) HM and (**c**,**d**) MHHM devices in log–log coordinates: (**a**) HM and (**c**) MHHM under positive-bias sweeps, (**b**) HM and (**d**) MHHM under negative-bias sweeps. Arrows mark the sweep direction. The colored straight segments are linear fits over the indicated voltage ranges, with the different colors distinguishing the low-field and high-field fitting regions of each branch as defined in the panel legends, and the fitted slopes (uncertainty within ±0.1) are identified in the text by panel and color. Because individual segments span limited voltage ranges, in some cases only a few data points, the slopes are used as qualitative indicators of the transport regime rather than as definitive mechanistic assignments, and segments containing only a few data points, including the steep Set transition itself, are excluded from the fitting.

The MHHM device exhibits an on/off ratio between approximately 10^2^ and 10^3^ depending on the read voltage, reaching up to approximately 10^3^ as read from Figure 2b and Figure 3c, which clearly outperforms the HM control under identical measurement conditions. The comparatively large HRS leakage of the HM device, which is the origin of its small effective window, can be traced to two factors. The first factor is the high density of interfacial defects, including dangling bonds and charge traps, at the Ag/HfO_2_ and HfO_2_/TiN boundaries, which provide trap-assisted transport paths. The second factor is the residual conductive paths left by incompletely ruptured filaments, which prevent the device from reaching a true HRS. Introducing the MoS_2_ interlayer mitigates both contributions. The atomically smooth van der Waals interface reduces dangling-bond-related traps and charge scattering, and the heterojunction regulates the vacancy migration path so that filaments form and rupture at reproducible and well-defined locations. The heterostructure thus acts as a nanoscale valve that guides vacancies along preferred paths instead of allowing random nucleation. In addition, sulfur-vacancy sites in MoS_2_ may serve as capture centers that anchor the filament formation site [34,36,47]. The consequence is a more uniform filament geometry from cycle to cycle, which suppresses both excessive filament growth and premature rupture.

To gain insight into the charge transport in the two structures, the current-voltage characteristics were replotted in log-log coordinates in Figure 3. Linear fits within the indicated voltage ranges are shown as colored segments, and the extracted slopes are used as indicators of the corresponding transport regime. The mechanistic meaning of the slope in such a double-logarithmic representation is well established [48,49,50]. A slope close to unity corresponds to Ohmic conduction through a continuous conductive path, a slope between 1 and 2 indicates trap-limited transport, a slope near 2 corresponds to the trap-filled (Child’s-law) regime of space-charge-limited current, still steeper segments reflect trap-filled-limit behavior associated with an exponential trap distribution, and sub-unity slopes indicate that transport is limited by interfacial barriers or by trap-assisted hopping rather than by band-like conduction. For the HM device under positive bias, shown in Figure 3a, two branches can be distinguished. Along the LRS branch, the slope is approximately 1.5 at lower voltage, indicating near-Ohmic but non-ideal conduction that is attributed to carrier scattering at interfacial defects superimposed on filamentary conduction. At higher voltage along the same branch, the slope decreases to approximately 0.35, a sublinear dependence consistent with trap-assisted transport through a partially connected filament network in which the current is limited by trap filling rather than by bulk migration. Along the HRS branch, the slope is approximately 2.2 at low field, consistent with trap-filled-limited transport over an exponential trap distribution [48,50], and decreases to approximately 0.66 at higher field as transport becomes limited by interfacial barriers and trap-assisted hopping, reflecting vacancy redistribution and progressive but incomplete filament connectivity. Under negative bias, shown in Figure 3b, the HRS and LRS curves nearly overlap, and the fitted slope increases from approximately 0.49 at low field to approximately 1.51 at higher field. This behavior is consistent with the picture developed above. Because the filaments in the HM device rupture incompletely during the preceding positive sweep, the device never reaches a well-defined HRS, and the negative sweep probes a residual and partially connected filament network whose conductance rises gradually without a sharp Set event.

The MHHM device shows markedly simpler behavior, as shown in Figure 3c,d. In the LRS, the fitted slopes remain close to unity in both polarities, with values of approximately 1.19 and 0.83 under positive bias and approximately 1.07 under negative bias, consistent with dominant Ohmic conduction through a well-formed filament, with only minor deviations attributable to defect scattering at the MoS_2_/HfO_2_ interface. Under negative bias, the pre-Set HRS branch exhibits a sub-unity slope of approximately 0.72, indicating that at low field the applied energy primarily drives vacancy migration and trap filling rather than band-like conduction, which constitutes a preparatory stage for the Set transition. The subsequent rise to the LRS is very steep, and this segment corresponds to the abrupt and threshold-like Set transition. It contains only a few data points and is therefore excluded from the slope analysis, as are all comparably short segments. Under positive bias, the device remains in the LRS with near-Ohmic behavior until the Reset event and afterwards settles onto a low-current branch with a sub-unity slope of approximately 0.33, corresponding to a stable and low-leakage HRS in which transport is again trap-assisted. This comparison suggests that the MoS_2_ interlayer promotes more complete filament rupture and suppresses residual conductive paths. Overall, the heterostructure extends and stabilizes the Ohmic LRS range and yields a cleaner Reset transition than the HM control.

**Figure 4:**
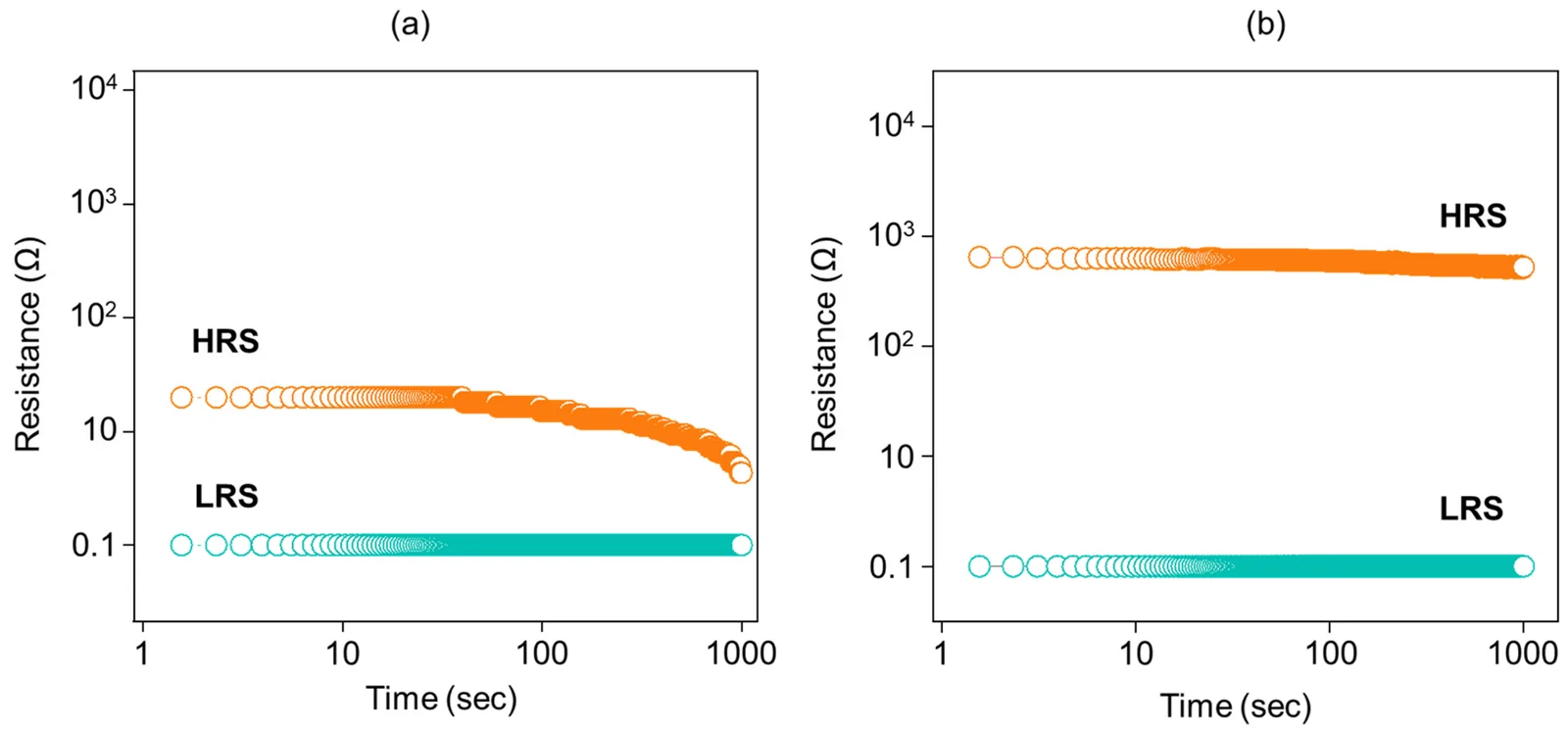
Retention characteristics of (**a**) HM and (**b**) MHHM devices, recorded at a read voltage of [0.1] V after Set/Reset programming. The read voltage was verified to cause no measurable disturbance of either resistance state.

The non-volatile behavior of both devices was evaluated by monitoring the HRS and LRS resistance under repeated readout at a small read voltage of [0.1] V following a single Set or Reset operation, as shown in Figure 4. The flat LRS trace of the MHHM device is not an artifact of current compliance, because the read current in the LRS stays well below the applied compliance current and varies from cycle to cycle, with the peak LRS current fluctuating between 2 × 10^−2^ A and 4 × 10^−2^ A at −1 V in Figure 2b. The retention trace therefore reflects the stability of the programmed state itself rather than compliance clipping. The HM device exhibits a pronounced decay of its HRS resistance, which drops by orders of magnitude within the 10^3^ s observation window, indicating poor state stability. This relaxation is attributed to the high density of interfacial defects at the HfO_2_/TiN boundary. Weakly confined vacancies slowly diffuse and re-aggregate near the ruptured filament, gradually restoring conductive paths and eroding the HRS, which is a typical thermally driven relaxation process. In contrast, both the HRS and the LRS of the MHHM device remain essentially flat over the same 10^3^ s window, with no measurable decay. Two factors plausibly contribute to this stability. The reduced interfacial defect density suppresses the slow vacancy diffusion that drives relaxation, and the MoS_2_ interlayer anchors the rupture location so that the filament gap reopens completely and at a reproducible position after each Reset [10,12]. Residual vacancies remain confined, which hinders the reformation of leakage paths. Under identical measurement and programming conditions, the contrast between the two devices within the 10^3^ s window is direct and unambiguous. We note that a 10^3^ s window is short compared with the 10^4^ to 10^5^ s demonstrated for optimized HfO_2_-based devices [27,45], so the present retention comparison is restricted to the measured window, and longer retention windows remain a necessary next step.

**Figure 5:**
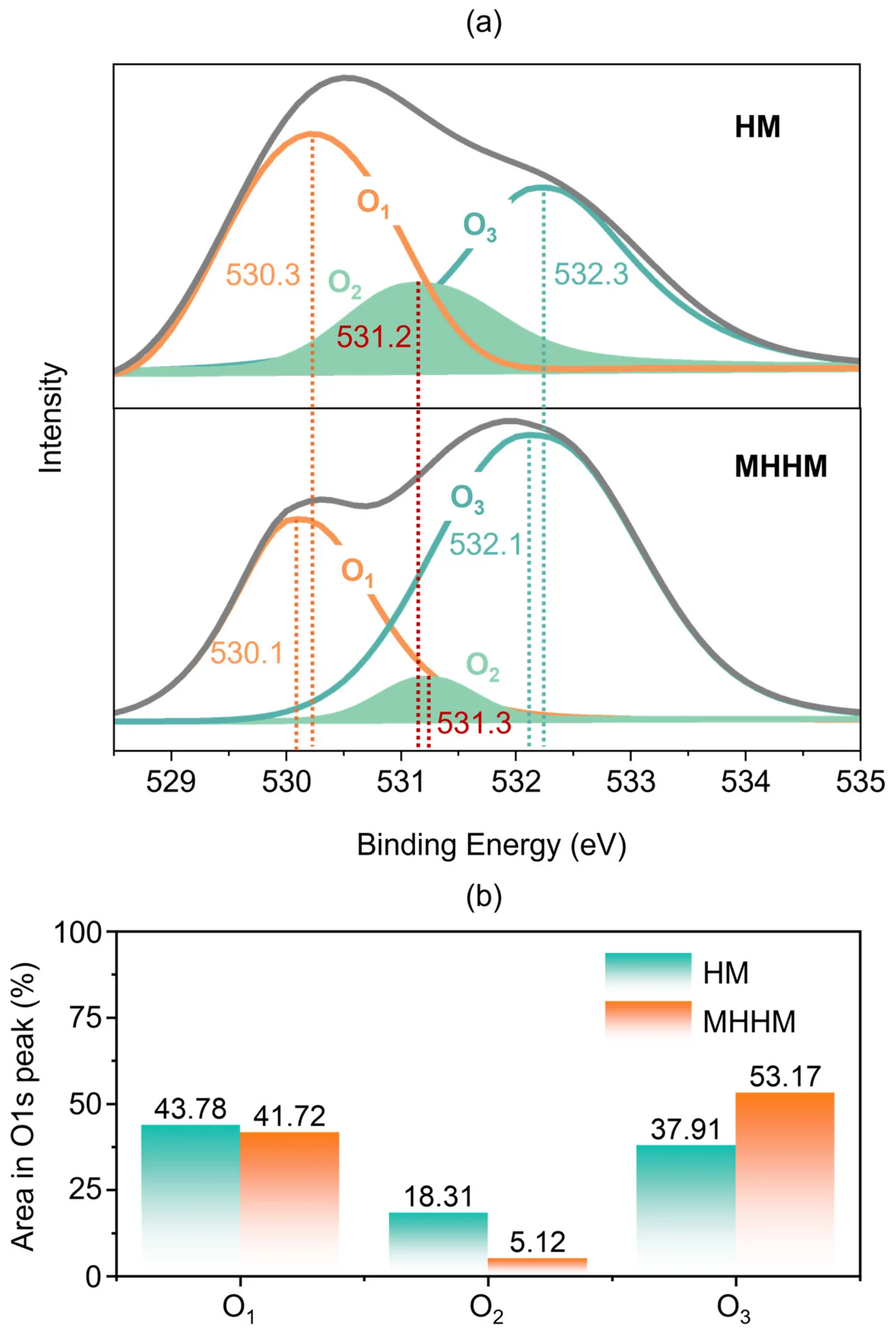
(**a**) XPS O 1s narrow-scan spectra of the HM (top) and MHHM (bottom) structures with three-component peak fitting: O_1_ (Hf–O lattice bonds, ~530.3/~530.1 eV), O_2_ (oxygen-vacancy-related defects, ~531.2/~531.3 eV), and O_3_ (surface-adsorbed oxygen/hydroxyl groups, ~532.3/~532.1 eV). (**b**) Relative areas of the three fitted components for the two devices. For the MHHM sample, the few-layer MoS_2_ overlayer attenuates all HfO_2_-related O 1s components by approximately the same factor, so the comparison is based on relative component areas, which are insensitive to uniform overlayer attenuation.

To probe the chemical origin of these differences, O 1s narrow-scan XPS spectra were recorded for both structures, as shown in Figure 5a. Three components are resolved in each spectrum. The O_1_ peak near 530.3 eV in HM and 530.1 eV in MHHM is assigned to Hf-O bonds of the HfO_2_ lattice. The O_2_ peak near 531.2 eV in HM and 531.3 eV in MHHM corresponds to oxygen-deficient and vacancy-related bonding environments that govern filament formation. The O_3_ peak near 532.3 eV in HM and 532.1 eV in MHHM is attributed to surface-adsorbed oxygen or hydroxyl species. The small shifts of 0.1 to 0.2 eV between the two samples are consistent with modified charge transfer at the MoS_2_/HfO_2_ interface, which alters the local electron density around the oxygen sites. XPS is inherently surface-sensitive, with an information depth of only a few nanometers, and for the MHHM sample the MoS_2_ overlayer additionally attenuates the photoelectrons originating from the buried HfO_2_. With an inelastic mean free path of approximately 2 to 3 nm for O 1s photoelectrons excited by Al Kα radiation, a few-layer MoS_2_ overlayer reduces the absolute intensity of the buried HfO_2_ signal by a factor of approximately exp(-d/λ). Crucially, however, such overlayer attenuation scales all HfO_2_-related O 1s components by the same factor and therefore leaves their relative areas unchanged. The extracted component ratios thus characterize the near-surface and near-interface region of the film, and since both samples were measured and fitted under identical conditions, the comparison between the two structures is carried out in terms of the relative component areas. The analysis focuses on the O 1s region because it probes the vacancy-related states most directly.

Within these constraints, the relative area of the vacancy-related O_2_ component decreases from 18.31% of the fitted O 1s envelope in the HM device to 5.12% in the MHHM device, a relative reduction of approximately 72%, while the O_1_ lattice component remains essentially unchanged (43.78% versus 41.72%) and the O_3_ surface-adsorbate component increases from 37.91% to 53.17%, as summarized in Figure 5b. Two aspects of this result warrant emphasis. First, because overlayer attenuation scales all HfO_2_-related components by the same factor, the selective suppression of the O_2_ component at nearly constant O_1_ cannot be explained by attenuation of the buried oxide signal under the MoS_2_ overlayer and must reflect a genuine modification of the oxygen bonding environment near the interface. Second, the near-constancy of the O_1_ lattice component indicates that the HfO_2_ lattice itself remains intact, which excludes decomposition or wholesale reduction of the switching layer as the origin of the O_2_ decrease. It should also be emphasized that the O_2_ component is a proxy for oxygen-deficient bonding environments, that is, oxygen ions adjacent to vacancy sites, rather than a direct count of vacancies, and that the assignment of O 1s components in the 531 to 532 eV range requires care [23,51]. Within this established assignment, the MoS_2_-terminated surface is proposed to passivate electrically active vacancy configurations at the MoS_2_/HfO_2_ interface. Interfacial sulfur can occupy oxygen-vacancy sites and saturate under-coordinated Hf cations, forming Hf–S-like bonding configurations whose oxygen neighbors no longer contribute a distinct O_2_ signature, consistent with the known interfacial chemistry of HfO_2_ on MoS_2_ [37]. The concomitant increase in the O_3_ component is attributed to stronger surface adsorption on the heterostructure. This passivation picture is consistent with all present observations, although direct chemical confirmation of Hf–S bonding would require dedicated core-level or depth-resolved analysis beyond the present comparative study. The device-level consequence is nevertheless clear. A smaller population of electrically active vacancies implies fewer competing nucleation sites, so that filaments form at a limited number of energetically preferred and interface-defined locations, consistent with the narrower Set and Reset voltage spread and the improved cycle-to-cycle overlap observed in Figure 2b.

**Figure 6:**
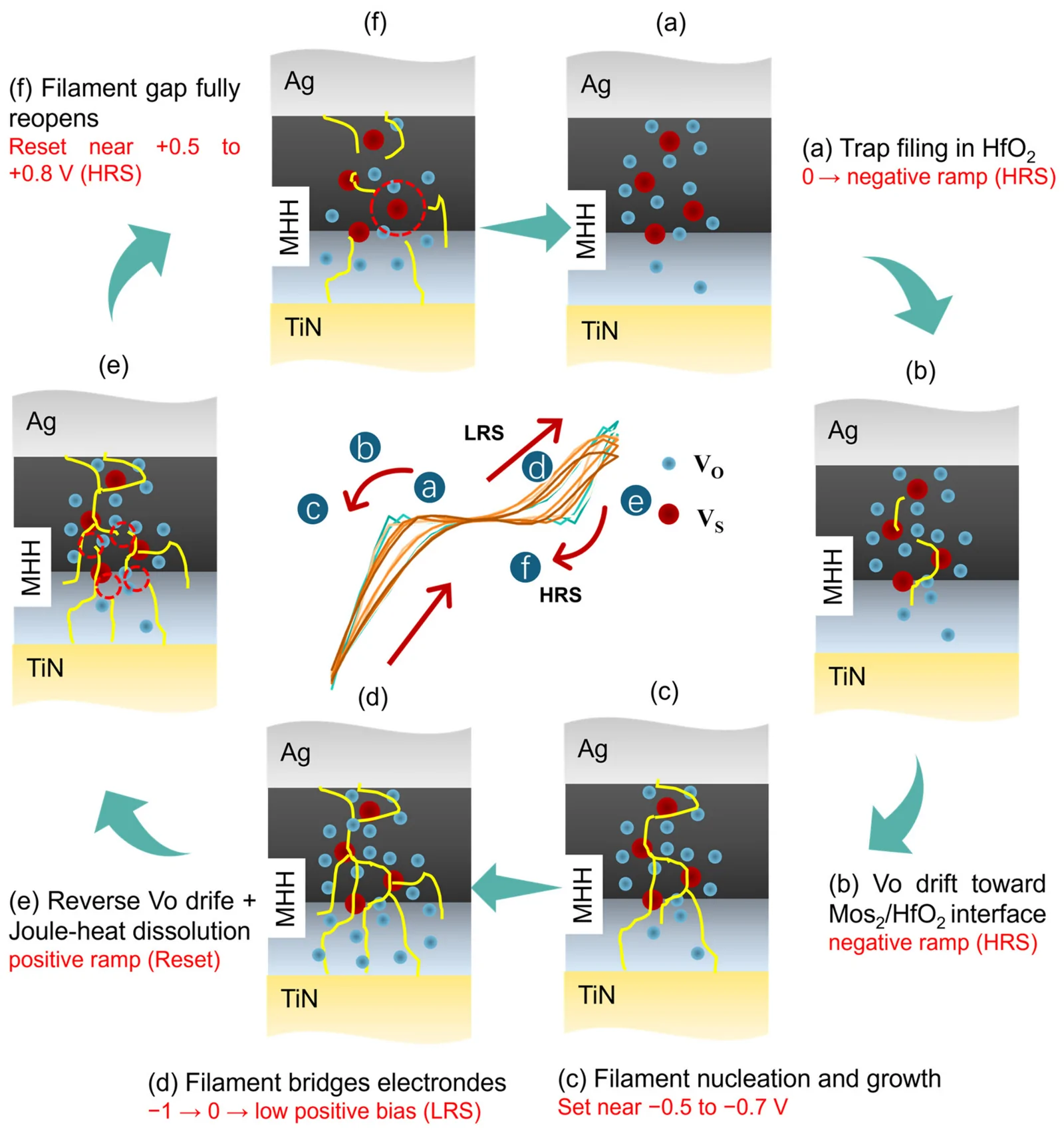
Schematic illustration of the proposed switching mechanism of the MHHM device. Stages (**a**–**f**) depict the evolution of the conductive filament during one full sweep, with the corresponding region of the I–V curve indicated for each stage: (**a**–**c**) negative-bias ramp, HRS → LRS (Set, near −0.5 V to −0.7 V); (**d**) low positive bias, LRS; (**e**,**f**) positive-bias ramp, LRS → HRS (Reset, near +0.5 V to +0.8 V).

Figure 6 illustrates the proposed switching mechanism of the MHHM device, with each stage mapped onto the current-voltage characteristics of Figure 2b. In the initial HRS under a low negative field, injected carriers progressively fill deep-level vacancy-related traps in the HfO_2_ layer, and transport is dominated by trap-assisted processes, consistent with the sub-unity slope observed in Figure 3d. As the negative field increases, the positively charged vacancies drift toward the negatively biased top electrode, that is, toward the MoS_2_/HfO_2_ interface. We propose that the sulfur-vacancy sites of the MoS_2_ layer provide uniformly distributed and energetically preferred accumulation sites, and that the atomically smooth van der Waals interface homogenizes the local field, which would reduce the randomness of nucleation. This interpretation is consistent with the XPS and electrical results presented above. Once the vacancy accumulation at the interface reaches a critical density, a vacancy-rich filament grows from the interface region toward the TiN bottom electrode and bridges the two electrodes, so that the device switches abruptly into the LRS. The conduction in this state is Ohmic, with slopes close to unity in Figure 3c,d. During the return ramp through 0 V into the low positive region, the filament remains intact and the device stays in the LRS. As the positive field increases further, the reversed field drives vacancies back toward the bottom electrode, while Joule heating assists dissolution of the filament at its thinnest point near the interface, and the current departs from linearity. Finally, the filament gap fully reopens near +0.5 V to +0.8 V, producing a steep current collapse, and the device returns to a stable and low-leakage HRS. The anchoring of the nucleation and rupture sites by the MoS_2_ interlayer makes this sequence repeatable from cycle to cycle and transforms the otherwise stochastic filament dynamics into a more controlled process.

Because the top electrode is electrochemically active Ag, electrochemical metallization, involving oxidation of Ag, migration of Ag ions, and reduction into a metallic filament, must be considered as a competing mechanism. Ag-filament formation has been demonstrated in MoS_2_-containing stacks [52,53], and mixed Ag-ion and vacancy conduction has been reported for TiN/HfO_2_/Ag devices [43,44], so this mechanism cannot be dismissed a priori. Several observations nevertheless argue that electrochemical metallization is not the dominant process here. First, the polarity is opposite to that expected for metallization. Injection of Ag ions from the top electrode requires a positive bias on Ag, whereas the Set transition occurs under negative bias on Ag, a polarity consistent with the drift of positively charged vacancies toward the top electrode in a valence-change picture [49]. Second, the switching uniformity correlates with the vacancy-related XPS signal rather than with any signature of metallic filaments. Third, the HRS of the MHHM device shows no measurable decay over 10^3^ s, whereas metallization-based devices frequently exhibit HRS degradation caused by undissolved metallic clusters. Regarding the role of MoS_2_ itself, the literature is nuanced, and vertically oriented or defective MoS_2_ can facilitate Ag-ion migration along van der Waals gaps and defect sites [52,53]. The MoS_2_ layer is therefore not claimed to be a perfect Ag barrier. In the present configuration, the basal plane of the exfoliated flake lies perpendicular to the field-driven ion path, which is expected to hinder, though not necessarily to block, direct Ag-ion injection into the HfO_2_ bulk. We therefore regard the interface-guided valence-change process as the dominant mechanism consistent with the present data, while acknowledging that a minor Ag-ion contribution cannot be excluded on the basis of these data alone.

A further question is whether the MHHM device should be regarded as two memristors in series, namely an Ag/MoS_2_ element stacked on an HfO_2_/TiN element, rather than as a single interface-engineered device. Three features of the data argue against the series picture. First, a series combination of two switching elements would be expected to produce two distinct switching events or, at minimum, threshold voltages reflecting the superposition of both elements, whereas the measured current-voltage curves exhibit a single sharp Set and Reset per cycle. Second, MoS_2_-based memristors reported to date switch predominantly by metal-ion mechanisms with the Set occurring under positive bias on the active electrode [34,52,53], which is the opposite polarity of what is observed here. Third, the switching behavior correlates with the vacancy-related O 1s signal of the HfO_2_ layer in Figure 5, indicating that HfO_2_ remains the active switching medium while the MoS_2_ layer modulates its interfacial defect landscape. These arguments support the interpretation of an interface-modulated single switching element.

## 4. Device Level Relevance to Intelligent Sensing

The electrical results establish a direct connection between interface engineering and the memory requirements of intelligent sensor nodes. The on/off ratio of up to approximately 10^3^ provides a readout margin for distinguishing two stored states. The reproducible Set and Reset transitions support repeated updating of a stored parameter, and the stable HRS and LRS retention over 10^3^ s allows information to persist between readout events without continuous refresh. These properties address different stages of the same local memory operation, namely writing, reading, and retaining a state.

In a robotic tactile node, a separate pressure or strain transducer would generate the sensory signal, while the present memristor could store a contact threshold, a calibration coefficient, or a compressed feature associated with a recent stimulus history. A wide resistance window would reduce ambiguity during state readout, and retention would preserve the selected state while the robot continues subsequent sensing operations. These assignments follow from the measured electrical characteristics and from the memory requirements of distributed robotic sensing [15,16]. They do not constitute experimental demonstrations of tactile recognition or robot control. Integration with sensor arrays and peripheral encoding circuits, together with longer retention and endurance evaluation, would be required to establish system-level sensing performance.

To place the present devices in the context of the state of the art, Table 1 compares their key switching metrics with representative HfO_2_-based and MoS_2_-containing resistive switching devices reported in the literature. The comparison shows that the present heterostructure devices operate at comparatively low voltages with a competitive on/off window and improved cycle-to-cycle reproducibility, while their demonstrated retention window remains below the best reported values.

## 5. Conclusions

Ag/MoS_2_/HfO_2_/TiN/SiO_2_/Si memristors incorporating a MoS_2_/HfO_2_ heterostructure were fabricated and compared with Ag/HfO_2_/TiN/SiO_2_/Si control devices processed under the same oxide deposition and annealing conditions. The heterostructure device exhibits reproducible bipolar switching with a single well-defined Set under negative bias and Reset under positive bias per cycle, an on/off ratio of up to approximately 10^3^, and markedly better cycle-to-cycle overlap than the control, whose switching window closes after repeated sweeps. O 1s XPS analysis shows that the vacancy-related O_2_ component decreases from 18.31% to 5.12% of the fitted envelope after introduction of the MoS_2_ interlayer, while the lattice-oxygen component remains essentially unchanged. This selective reduction is inconsistent with a pure attenuation artifact and is interpreted in comparative and surface-sensitive terms as evidence of interfacial passivation of electrically active vacancies rather than bulk oxygen extraction. Retention measurements over 10^3^ s show stable high- and low-resistance states for the heterostructure device, in contrast to the strong HRS relaxation of the control. The switching polarity, the vacancy-chemistry correlation, and the stability of the ruptured state are consistent with an interface-guided valence-change mechanism, while not definitively excluding a minor Ag-ion contribution. We emphasize the limitations of the present study. The control device did not undergo the transfer-related N_2_ annealing step, the demonstrated retention window of 10^3^ s is shorter than state-of-the-art values for HfO_2_-based devices, and device-to-device statistics, endurance, and temperature- and time-resolved characterization remain to be completed. The demonstrated switching window, repeatability, and retention establish the relevance of this interface engineered device to nonvolatile memory in intelligent sensor nodes, including local storage of thresholds and features for robotic tactile perception. Future integration with transducers and peripheral circuits will be necessary to evaluate sensing performance at the system level. Extending these aspects will be the subject of subsequent work, toward establishing MoS_2_/HfO_2_ heterojunction engineering for oxide-based resistive memory in intelligent sensing hardware.

## Figures and Tables

**Table 1:** Benchmark comparison of the present Ag/MoS_2_/HfO_2_/TiN devices with representative HfO_2_-based and MoS_2_-containing resistive switching devices.

Device Structure	V_Set (V)	V_Reset (V)	On/Off Ratio	Endurance (Cycles)	Retention (s)	Ref.
Ag/ZrO_2_/MoS_2_/Pt	+0.8	−0.2	10^6^	10^2^	10^4^	[39]
Au/HfO_2_/Al-ZnO/HfO_2_/ITO	+0.65	−1.34	10^4^	10^2^	10^4^ (85 °C)	[45]
Fe_3_O_4_ nanocrystals/Ge nuclei/Si	n.r.	n.r.	≈58 (off/on)	n.r.	n.r.	[26]
Ag/MoS_2_/HfO_2_/TiN (this work)	−0.5 to −0.7	+0.5 to +0.8	up to ≈10^3^	repeated DC sweeps	10^3^	This work

## Data Availability

Data will be made available on request.
